# In the absence of light, the Nursing Now lighthouse illuminates the future*

**DOI:** 10.1590/1518-8345.0000.3356

**Published:** 2020-10-19

**Authors:** Isabel Amélia Costa Mendes, Maria Auxiliadora Trevizan

**Affiliations:** 1 Universidade de São Paulo, Escola de Enfermagem de Ribeirão Preto, PAHO/WHO Collaborating Centre for Nursing Research Development, COFEn collaborator-Coordinator of the GT Nursing Now Brazil, Ribeirão Preto, SP, Brazil.; 2 Universidade de São Paulo, Escola de Enfermagem de Ribeirão Preto, PAHO/WHO Collaborating Centre for Nursing Research Development, Ribeirão Preto, SP, Brazil.

**Figure:**
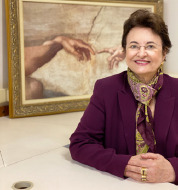


**Figure:**
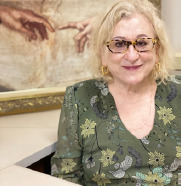


We are on the path to the emancipation of Nursing.

Since the beginning of our work in this profession, more than 50 years ago, we realized its context of unlimited subordination to and acceptance of other professionals’ orders - especially those of physicians.

During some of that time, at the beginning of our career, we witnessed facts that showed that many nurses not only agreed with this situation but also led their peers and nursing students to take actions that harmonized their performance in health services based on this servitude.

Clearly, we also felt that a few nurses, even though they fell under those circumstances, were not comfortable in the face of such intense dependence. Sometimes we witnessed conflicts and even revolt on the part of those who felt dominated. On the other hand, few professionals sought to show the importance of their work in the field of health, in addition to expressing their convictions about the need to acknowledge interdependence among other professionals. In turn, nurses showed ease in adjusting to the strict observance of organizational determinations, discipline, submission to rules and routines, to the point of transforming them from a means to an end in themselves, causing stiffness and excessive formalism in their actions^(^ 1 ^)^.

We considered this context of Nursing to be traumatic: this is why we say that an event can induce trauma if it causes excessive concern, if it disregards beliefs and values, and violates expectations toward a profession, stifling cognitive resources and causing inappropriate emotional responses. With this cognitive restriction and behavioral fragility, professionals’ performance was less than the sum of their skills and they faced difficulties when making decisions.

We have caught glimpses of changes in these dynamics.

Nonetheless, despite progressive changes, mainly in teaching, along with scientific and technological development, and the creation and development of specialties, not only does society fails to acknowledge the value of nurses, but it is also the case that very little has changed in the behaviors and attitudes of nurses in relation to their self-worth, assuming leadership positions and developing their political engagement. Considering the need for this profession and its social relevance, nurses must increase their sense of self-worth and expand their roles.

So far, the role of nurses remains incomprehensible. This obscurity and inability to perceive the qualification and attributes that enable the work of these professionals originate in large part from the health staff itself, including the nurses themselves and, therefore, society. It is, however, shocking to realize that, after so much time, the professional preparedness of nurses remains underutilized. The underutilization of nurses, something first reported almost four decades ago^(^ 2 ^)^, is still real and is now pointed out by respected and notable leaders^(^ 3 ^)^. Our conception, shared by other authors^(^ 4 ^-^ 5 ^)^, is that the efficiency and effectiveness of nurses remain hidden, invisible, not fully appreciated by the community.

Hence, as a result of this lack of light and of vision and intelligibility from the social body toward Nursing, the profession is permeated with a lack of prestige. Society has ignored and failed to acknowledge the legitimacy of the merit of and need for nurses: conferring upon them a condition of an almost unknown profession, not fully realizing the essence and excellence of the nurses’ practice within the field of health. Due to a lack of clarity, both health workers and society as a whole fail to appreciate and fairly recognize the work nurses perform.

The distinctiveness of the relevant work performed by nurses urgently requires acknowledgment^(^ 6 ^)^!

This topic has been paramount in the documents supporting the Nursing Now Campaign^(^ 7 ^-^ 8 ^)^, which address dilemmas and challenges that mobilized leaders of governments and international organisms to take converging actions, based on a finding that without nurses and midwives, there will be no foundation and functional structure to provide integral health care, because both are essential to the implementation of sustainable development and universal health coverage. From this perspective, we conclude that overcoming this obstacle requires investment in education and policies that translate into improved work conditions, decent wages, respect, autonomy and the adoption of strategies able to overcome scarcity, encouraging vocations and attracting young talent, while preserving valuable human resources who have already chosen this profession and deserve recognition.

The forecast is that existing health services will only be kept if the workforce, currently numbered at 27.9 million nursing and midwifery professionals^(^ 9 ^)^, receive investment and support to retain, renew and expand to by least 4.7 million individuals in the next ten years. Note that this expansion was estimated in the context of a relatively stable scenario, upon completion of data collected for the State of the World’s Nursing Report^(^ 9 ^)^. Nonetheless, given devastating turbulence caused by COVID-19, whereas the loss of life significantly affects nursing (and health) workers and students, as well, this view of the deficit is already outdated, despite the very recent launching of this first report^(^ 9 ^)^ on April 6^th^, 2020 by the World Health Organization , International Council of Nurses (ICN), and Nursing Now Campaign.

The paradigm shift that is envisaged with the Nursing Now Campaign requires evidence to convince governments and employers to invest more, with better quality investment, in human resources in the nursing field and adopt inclusive policies at the governmental and organizational levels, both in the public and private sectors. Therefore, we should make room for nurses to have an active voice in the formulation of health policies, rather than merely ensure and guarantee the implementation of health policies that are invariably credited to other professionals, so nurses start having an assured position at deliberative tables. In this way, nurses will be included and heard, showing their contribution at the political and decision-making level, making use of their vision and experience to draw up strategic and operational definitions that are appropriate and feasible for each context.

In line with international organizations, this new concept of value is expected to be reflected in the recruitment of nurses for leadership positions.

We know leadership makes all the difference!

For those aware and inspired nurses to acquire skills and better understanding, and improve the development of their leadership potential, they should consider their unique characteristics, recognize their value, and acknowledge the need to expend effort to assume and fulfill certain requirements and conditions to reach a leadership position. Once this position is attained, be always vigilant and continually improve competencies and knowledge already acquired, while concomitantly dedicating time to train new leaders. Mobilizing this leadership into continuous improvement is part of a leader’s role as the representative of Nursing to the decision-making body of the organization with which one is affiliated. It is in this sphere that nurses will present proposals intended to establish policies and strategies of continuous personal development in search for quality, innovation, and autonomy. In this same instance, nurses will present results and the impact of investment in quality, showing the efficacy of nursing services in care for patients and also efficiency in the involvement of workers. In doing so, other professionals will provide support, triggering inter-professional projects, and contributing to an innovative culture in work processes, in a positive organizational environment that values its components. By provoking this process of change, setting in motion and acting as an activator of projects, programs, and processes, this leader-nurse and her/his supporters will project a new professional image that reflects upon the entire organization, inspiring peers to reproduce such behavior in other health settings, little by little, disseminating it to the whole of society.

Purposes such as these, in all the sectors of nursing practice and at different situations, are extremely valid and welcomed to strengthen individuals and the collective, empowering the profession and the workers therein. The validation of such purposes, however, depends on legitimacy associated with an official position that has power, such as recommended by the International Council of Nurses and the World Health Organization: the position of Government Chief Nursing Officer, at the highest level of Ministries of Health.

And here is our challenge: why is it that we still do not have Government Chief Nursing Officer in some countries?

The main answer is an absence of leadership, which directly results in political obstacles that can only be overcome to the extent that we collectively develop strong leadership capable of convincing Ministries of Health to follow the WHO’s recommendations.
